# Role of bioinformatics in establishing microRNAs as modulators of abiotic stress responses: the new revolution

**DOI:** 10.3389/fphys.2015.00286

**Published:** 2015-10-26

**Authors:** Anita Tripathi, Kavita Goswami, Neeti Sanan-Mishra

**Affiliations:** Plant Molecular Biology Group, International Centre for Genetic Engineering and BiotechnologyNew Delhi, India

**Keywords:** microRNA, abiotic stress, high-throughput sequencing, microarray, bioinformatic approached, degradome, NGS

## Abstract

microRNAs (miRs) are a class of 21–24 nucleotide long non-coding RNAs responsible for regulating the expression of associated genes mainly by cleavage or translational inhibition of the target transcripts. With this characteristic of silencing, miRs act as an important component in regulation of plant responses in various stress conditions. In recent years, with drastic change in environmental and soil conditions different type of stresses have emerged as a major challenge for plants growth and productivity. The identification and profiling of miRs has itself been a challenge for research workers given their small size and large number of many probable sequences in the genome. Application of computational approaches has expedited the process of identification of miRs and their expression profiling in different conditions. The development of High-Throughput Sequencing (HTS) techniques has facilitated to gain access to the global profiles of the miRs for understanding their mode of action in plants. Introduction of various bioinformatics databases and tools have revolutionized the study of miRs and other small RNAs. This review focuses the role of bioinformatics approaches in the identification and study of the regulatory roles of plant miRs in the adaptive response to stresses.

## Abiotic stresses and their impact on yield

Plants are exposed to a wide array of environmental fluctuations that lead to various physiological and metabolic changes, which in turn adversely affect the growth and productivity. Abiotic stresses are the principal cause of decrement in crop production globally and are responsible for lowering the average yield of major crops by more than 50% (Mahajan and Tuteja, 2005; Rodríguez et al., 2005). The World Meteorological Organization has reported that the years from 2001 to 2010 were considered to be the warmest period after 1850 (Oosterhuis, 2013). The climate change models have predicted that in coming time the occurrence and severity of such stresses will increase, leading to a decrease in agricultural production by about 70% (Cramer et al., 2011; Hasanuzzaman et al., 2013c; Ghosh and Xu, 2014).

**Figure 1:**
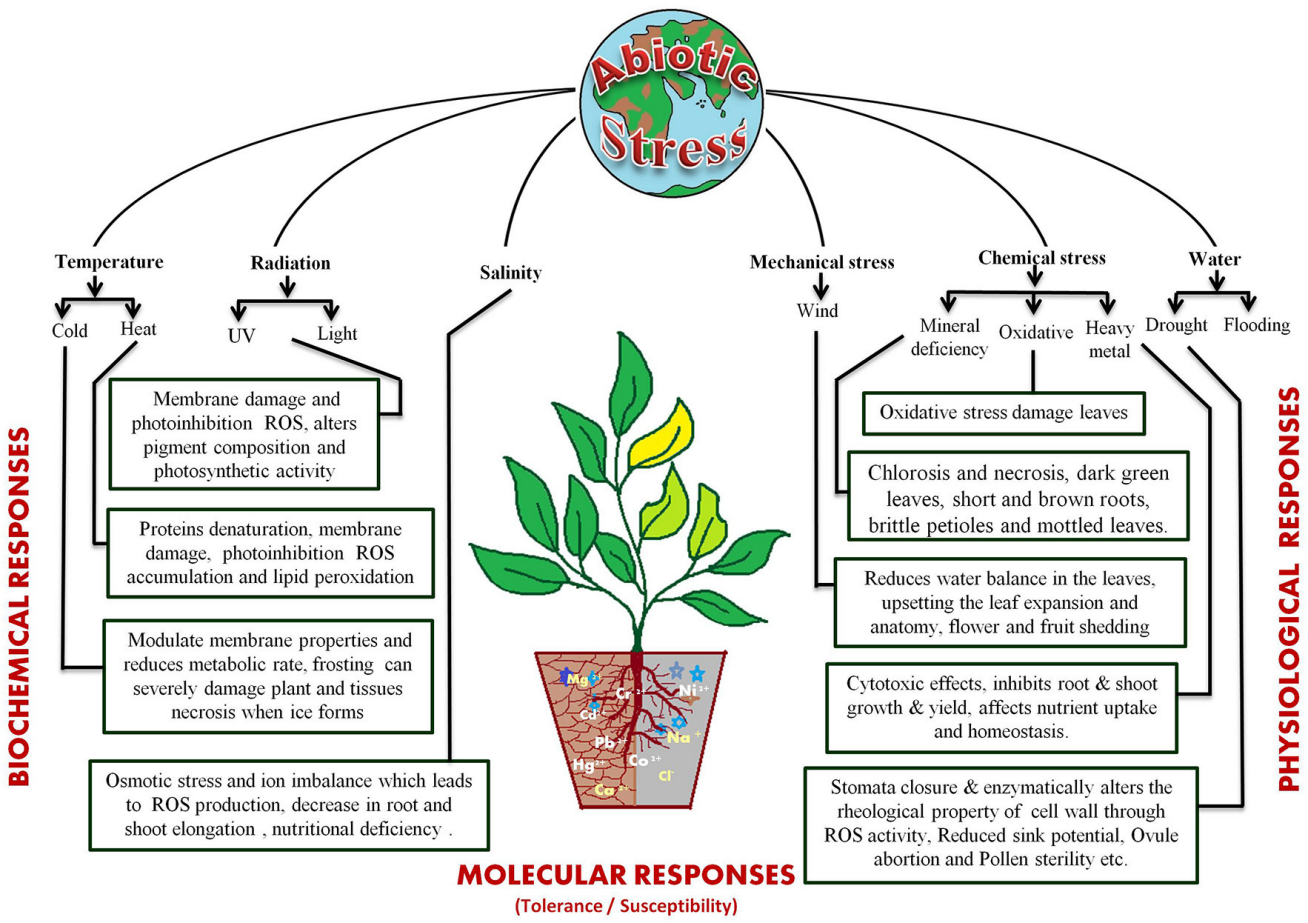
**Various abiotic stresses and their physiological effects on plants**.

The different abiotic stress conditions may be segregated into 35 different types that can be sorted as 11 groups, viz. cold, heat, drought, flooding, radiations (UV and light), wind, salinity, heavy metal toxicity, nutrient deprivation in soil, and oxidative stress (Mahajan and Tuteja, 2005). These stresses act by affecting plant growth at the molecular, biological, and physiological levels (Figure 1). The most studied abiotic stress conditions are cold, high temperature, salt, and drought stress. Plants cannot escape from these stresses because of their sessile nature but, they have developed sophisticated systems to cope up with them (Nakashima et al., 2009; Pfalz et al., 2012; Upadhyaya and Panda, 2013). The response to abiotic stresses is usually multigenic and involves altering the expression of nucleic acids, proteins and other macromolecules (Figure 1). Several excellent reviews are available that discuss the impact of these stresses on plants in details (Cramer et al., 2011; Shanker and Venkateswarlu, 2011; Duque et al., 2013; Hasanuzzaman et al., 2013a; Rejeb et al., 2014; Petrov et al., 2015; Sodha and Karan, 2015).

Primarily fluctuations in available water, temperature and soil salt content are recognized as the basic environmental stress factors. The scarcity of water because of less rainfall, paucity of soil water and excessive evaporation, is probably the most common factor, limiting the crop's growth (de Oliveira et al., 2013). Water deficit negatively affects plant growth and development by modulating nutrient uptake, photosynthesis, hormonal levels, water potential etc. This often results in tissue dehydration leading to senescence (Kaiser, 1987; Aroca et al., 2001, 2012; Kacperska, 2004; Wahid and Close, 2007). Under low water conditions plants activate their protective machinery to enhance water uptake and reduce water loss. However, deficiency of sufficient water supply or drought limits the root hydraulic conductivity (Nobel and Cui, 1992; North and Nobel, 1997; Aroca et al., 2012) thereby affecting water uptake and resulting in physiological drought condition for the plant (Bréda et al., 1995; Duursma et al., 2008; Aroca et al., 2012). Similarly, when the water level goes above the optimal levels it results in flooding which causes hypoxic conditions, stimulate the reactive oxygen species (ROS) and induces ethylene production that restricts aerobic respiration (Bailey-Serres and Voesenek, 2008; Perata et al., 2011).

Fluctuations in atmospheric temperature due to climate change are also exerting an adverse affect at physical and cellular levels. High temperatures change the cellular state, lipid composition, membrane fluidity, and organelle properties. They induce oxidative stress and reduce the water content of the soil, causing physiological drought in plants (Wahid and Close, 2007; Giri, 2011; Hasanuzzaman et al., 2013b; Goswami et al., 2014). They also affect flowering by decreasing the number of flowers, reducing pollen viability and flower fertility (Matsui et al., 2000; Prasad et al., 2000, 2006; Suzuki et al., 2001) and cause embryo damage during the early stages of seed germination (Grass, 1994; Hasanuzzaman et al., 2013b). Low temperatures also confer osmotic and oxidative stress on plants (Chinnusamy et al., 2007; Aroca et al., 2012). They reduce metabolic rate, increase rigidification of the cellular membrane, cause flower abortion, fertilization breakdown and negatively impact seed filling (Thakur et al., 2010; Zinn et al., 2010; Hedhly, 2011).

The temperature increases along with poor irrigation practices increase soil salinity. This has emerged as an important stress which inhibits plant's growth at every stage by inducing osmotic stress and ion toxicity (Diédhiou and Golldack, 2006; Joseph and Mohanan, 2013; Roychoudhury and Chakraborty, 2013). Salinity majorly affects roots by decreasing water use efficiency and ion exclusion, which adversely affects the root elongation, spike development and plant height (Choi et al., 2003; Alam et al., 2004; Diédhiou and Golldack, 2006; Mahmood et al., 2009; Aroca et al., 2012; Hakim, 2013; Pierik and Testerink, 2014).

The various environmental stresses result in osmotic and oxidative stresses, which inhibit metabolic reactions (Chinnusamy et al., 2007). Oxidative damage is one of the main reasons for loss of productivity and is triggered by increase in reactive oxygen species (ROS) that includes superoxide radicals (2O^−^), hydroxyl radicals (OH), and hydrogen peroxide (H_2_O_2_) (Mittler, 2002; Apel and Hirt, 2004; Bartels and Sunkar, 2005; Foyer and Noctor, 2005; Addo-Quaye et al., 2009). The ROS are responsible for nucleic acid damage, protein oxidation, and lipid peroxidation (Foyer et al., 1994). Plants have developed intrinsic mechanisms to avoid the oxidative stresses that includes recruitment of enzymatic scavengers, like superoxide dismutase (SOD), ascorbate peroxidase, glutathione peroxidase, glutathione S-transferase, catalase, and non-enzymatic low molecular mass molecules, such as ascorbate, tocopherol, carotenoids, and glutathione (Mittler, 2002; Mittler et al., 2004).

## Basics of microRNA

The discovery of regulatory small RNAs (sRNAs) that block specific messenger RNAs (mRNAs) at the post-transcriptional levels (PTGS or post-transcriptional gene silencing) by cleavage or translational repression (Sunkar et al., 2006; Shi et al., 2012) or interfere with transcription (TGS or transcriptional gene silencing) by directing DNA methylation of genes (Wu and Zhang, 2010) have unlocked a new avenue in gene expression regulation. The sRNAs constitute a large family represented by many species of RNA molecules distinguished from each other by their size, biogenesis, mode of action, regulatory role etc. (Axtell and Bowman, 2008; Sanan-Mishra et al., 2009; Lima et al., 2011; Meng et al., 2011a; Zheng et al., 2012).

The microRNA (miR) represents a major sub-family of endogenously transcribed sequences, ranging in length from 21 to 24 nt (Carrington and Ambros, 2003; Eldem et al., 2013). They have been established as a major regulatory class that inhibits gene expression in a sequence-dependent manner. The *lin-4* and *let-7* regulatory RNAs are accepted as the naissance member of the miR family (Lee et al., 1993; Reinhart et al., 2002), which is conserved across animal and plant species. Though there is no conservation between the animal and plant sequences, but high conservation is observed among plant miRs (Reinhart et al., 2002). An exception is provided by Ath-miR854 and Ath-miR855, which regulate levels of transcript encoding the oligouridylate binding protein 1b (UBP1b) (Arteaga-Vázquez et al., 2006). The target transcript of miR854 performs similar functions in plants as well as in animals (Arteaga-Vázquez et al., 2006).

### microRNA biogenesis

Each miR arises in the nucleus from an independent transcription unit, comprising of its own promoter, transcribing region and terminator, by utilizing the basic machinery for DNA-dependent RNA polymerase II mediated transcription (Kurihara and Watanabe, 2004; Lee et al., 2004; Xie et al., 2005a; Kim et al., 2011). Plant miR genes are present throughout the genome, although majority of the loci in plants are generally found in genomic (intergenic) regions that are not protein coding (Jones-Rhoades et al., 2006; Wahid et al., 2010). Comparatively lesser number of plant miRs are present in the introns (Lagos-Quintana et al., 2001; Lau et al., 2001; Chen, 2008; Nozawa et al., 2010; Wahid et al., 2010) and are rarely found in the exons (Olena and Patton, 2010; Li et al., 2011). Two miRs, miR436, and miR444, were mapped to the exonic regions of the protein-coding genes J023035E19 (AK120922) and J033125N22 (AK103332), respectively (Sunkar et al., 2005). It is hypothesized that the miRs control the host gene expression via a negative feedback loop mechanism that affects alternative splicing and cytoplasmic movement of transcripts (Slezak-Prochazka et al., 2013). Recently, CDC5 was identified as a MYB-related DNA binding protein that positively regulates miR production (Zhang et al., 2013a) by binding to their promoters and through interaction with the RNase III enzyme DCL1 (Dicer-Like 1). The large pri-miRs (primary transcripts) contain a 5′-cap and 3′-polyA tail and are stabilized in the nucleus by DDL (Dawdle) which is a RNA binding protein (Yu et al., 2008).

The pri-miRs are further processed into hairpin loop structured pre-miRs (precursor miRs) in the D bodies (Dicing bodies) or SmD3-bodies (small nuclear RNA binding protein D3 bodies) (Kurihara et al., 2006; Fang and Spector, 2007; Fujioka et al., 2007) by a protein complex containing the DCL1 (Schauer et al., 2002) and the CBC (Cap-Binding protein Complex) (Kim et al., 2008). The accuracy of DCL1 mediated pri-miR processing is promoted by both HYL1 (Hyponastic Leaves 1), and the C_2_H_2_-zinc finger protein, SE (Serrate) (Kurihara et al., 2006; Dong et al., 2008; Manavella et al., 2012a). This activity is also aided by DRB (Double strand RNA-Binding) protein (Han et al., 2004; Kurihara et al., 2006; Vazquez, 2006). Recently the G-patch domain protein TGH (Tough) was identified as another active player which is responsible for enhancing the DCL1 activity (Ren et al., 2012). It has been shown that HYL1 binds double stranded (ds) region on the pri-miR (Hiraguri et al., 2005; Rasia et al., 2010; Yang et al., 2010), TGH binds the single-stranded (ss) RNA region (Ren et al., 2012) and SE possibly binds at ssRNA/dsRNA junctions (Machida et al., 2011). It was also observed that HYL1 is a phospho-protein that directly interacts with CPL1 (C-terminal domain Phosphatase-Like 1) protein, to maintain its hypo-phosphorylated state (Manavella et al., 2012a). Thus, CPL1 also plays a critical role in accurate miR processing though it is not directly required for DCL1 activity (Manavella et al., 2012a). It was observed that CPL1 directly interacts with SE and a mutation in SE can affect phosphorylation status of HYL1 by preventing recruitment of CPL1 (Manavella et al., 2012a). Thus, the proposed model for the pri-miR processing indicates association of multiple RNA binding proteins with definite regions to maintain the structural determinants for recruiting and directing DCL1 activity. The DCL1, HYL1, SE, and TGH seem to interact directly (Kurihara et al., 2006; Lobbes et al., 2006; Yang et al., 2006; Qin et al., 2010; Machida et al., 2011; Ren et al., 2012) and are colocalized in the D bodies as shown by bimolecular fluorescence complementation. However, it has not been demonstrated whether they represent a stable plant microprocessor complex (Fang and Spector, 2007; Fujioka et al., 2007; Song et al., 2007; Manavella et al., 2012b; Ren et al., 2012).

The hairpin looped pre-miRs thus formed are further processed by DCL1 to produce miR/miR^*^ duplex (Xie et al., 2005b; Sanan-Mishra et al., 2009; Naqvi et al., 2012). Recently a proline-rich protein, SIC (Sickle), was identified to co-localize with HYL1 foci (Zhan et al., 2012) and it was found to play an important role in the accumulation of mature miR duplex (Zhan et al., 2012). The strands of the duplex are protected from uridylation and degradation by the activity of a methyltransferase protein known as HEN1 (Hua Enhancer 1) which covalently attaches a methyl residue at the 3′ ribose of last nucleotide from each strand (Li et al., 2005a; Yu et al., 2005). The miR duplexes are transported to the cytoplasm by HST (Hasty), the ortholog of Exportin-5 (Park et al., 2005), where the miR strand guides the AGO1 (Argonaute 1) containing RNA-induced silencing complex (RISC) complex to the target transcript (Baumberger and Baulcombe, 2005; Qi et al., 2005).

### microRNA function

Plant miRs generally control the expression of their targets transcripts by cleavage and translational repression (Chen, 2009). Brodersen et al. concluded that central matches in miR:target-mRNA duplex tend to cleave target mRNA, regardless of a few mismatches in other regions, while central mismatches in miR:target mRNA duplex lead to translational repression (Brodersen et al., 2008). It was hypothesized that the rapid fine-tuning of the target transcripts by translation repression is required for the reversible modulation of the negative regulators of stress responses whereas the on-off switching of target gene expression by cleavage was important in regulating developmental processes, which require permanent determination of cell fates (Baumberger and Baulcombe, 2005).

In plants, miRs regulate various biological processes such as, growth and development, pattern formation, organ polarity, signal transduction, and hormone homeostasis etc. (Palatnik et al., 2003; Dugas and Bartel, 2004; Jones-Rhoades et al., 2006; Mallory and Vaucheret, 2006; Mishra and Mukherjee, 2007; Cai et al., 2009; Voinnet, 2009; Sanan-Mishra et al., 2013). In past few years the role of miRs in response to diseases and environmental stresses has been highlighted (Fujii et al., 2005; Sunkar et al., 2007; Zhou et al., 2010; Lima et al., 2011; Meng et al., 2011a; Zheng et al., 2012; Mittal et al., 2013; Sharma et al., 2015). These are supported by reports on mutants of the miR biogenesis or pathways exhibiting defective phenotypes (Laufs et al., 2004; Zhong and Ye, 2004; Millar and Gubler, 2005; Ori et al., 2007; Chen et al., 2010; Rubio-Somoza and Weigel, 2011). The stress regulated miRs may be engaged in many biological pathways that re-program intricate procedures of physiology and metabolism (Khraiwesh et al., 2012) as suggested by their differential expression patterns in tissues in presence or absence of stress (Covarrubias and Reyes, 2010).

## Identification of stress-associated microRNAs

**Figure 2:**
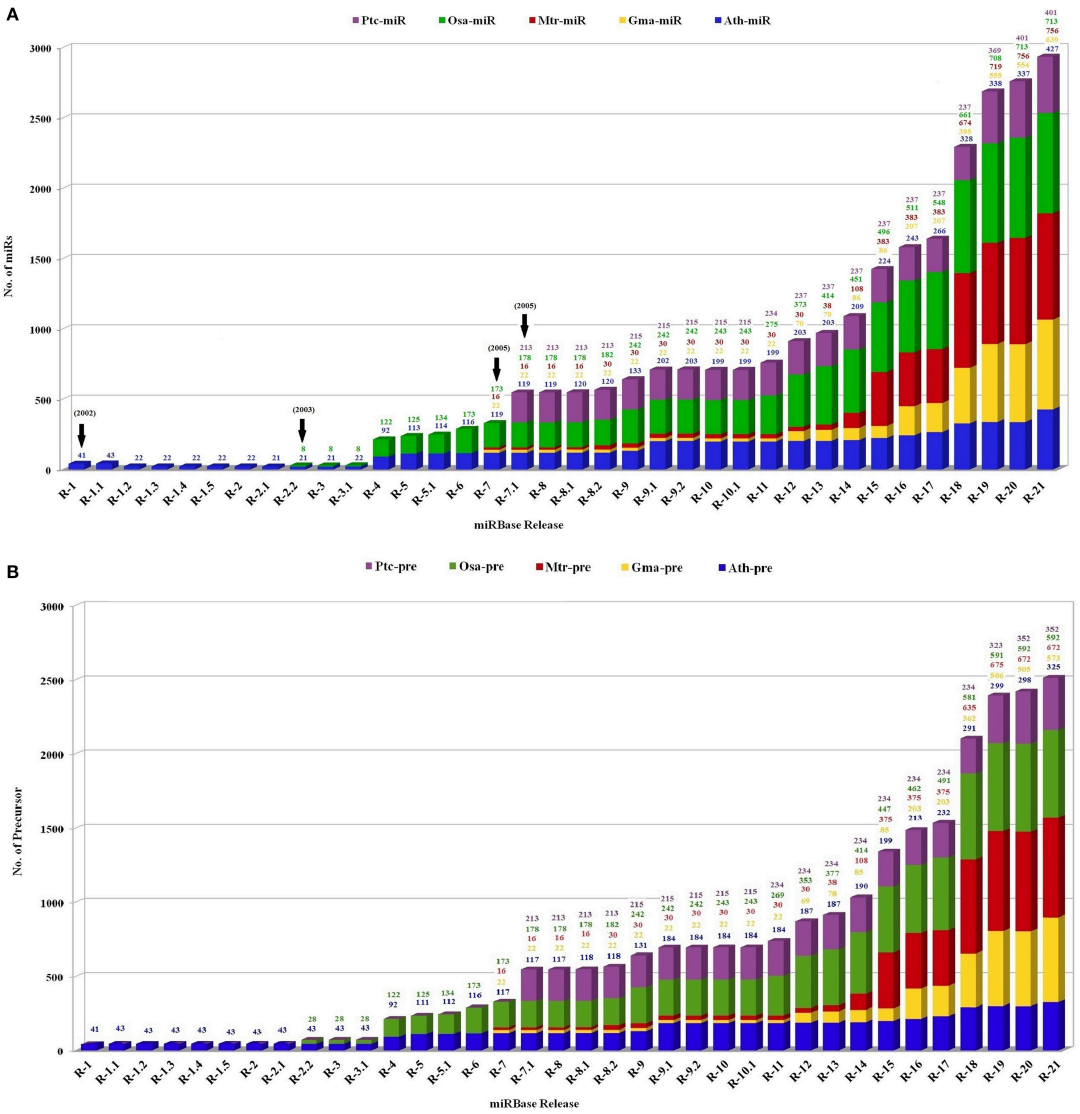
**The status of the number of (A) mature miRs and (B) precursor miR sequences in the miRBase registry in five plant species**. The plot contains the number of miRs in *Medicago truncatula* (red), *Oryza sativa* (green), *Glycine max* (yellow), *Populus trichocarpa* (violet), and *Arabidopsis thaliana* (blue) calculated with respect to total number reported miRs. X axis represents number of respective sequences and Y axis denotes the released versions of miRBase.

The identification of plant miR families began in the year 2000, with direct cloning and sequencing (Llave et al., 2002; Park et al., 2002; Reinhart et al., 2002). However, this was an uphill task owing to their small size, methylation status and multiple occurrences in genome. The numbers however increased rapidly with the advancement in cloning techniques and computational algorithms. In the past few years high throughput sequencing and screening protocols has caused an exponential increase in number of miRs, identified and functionally annotated from various plant species (Rajagopalan et al., 2006; Fahlgren et al., 2007; Jagadeeswaran et al., 2010; Rosewick et al., 2013). This is best exemplified by the establishment of miRBase, a biological database that acts as an archive of miR sequences and annotations (Griffiths-Jones, 2004; Griffiths-Jones et al., 2008; Kozomara and Griffiths-Jones, 2014). The first release of miRBase in the year 2002 included total 5 miRs from only 1 plant species, *Arabidopsis thaliana*. This was followed by the inclusion of *Oryza sativa*, in miRBase in the year 2003. Thereafter miRs reported from *Medicago truncatula, Glycine Max*, and *Populus trichocarpa* were included in the year 2005. The current version (release 21) includes 48,496 mature plant miRs derived from 6992 hairpin precursors reported in 73 plant species (Figure 2).

**Figure 3:**
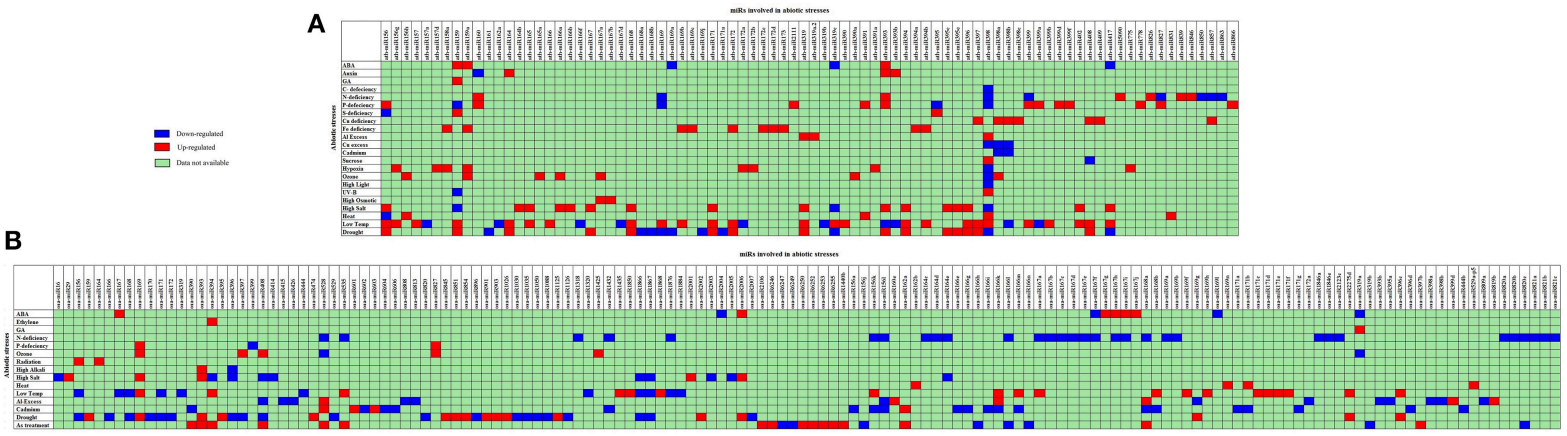
**Status of abiotic stress regulation for conserved miRs as reported from (A) Arabidopsis and (B) rice**. The X axis contains the list of abiotic stress regulated miRs, and Y axis lists the different abiotic stress studied. ABA, Abscisic Acid; GA, Gibberellic Acid; C, carbon; N, nitrogen; P, Phosphorus; S, Sulfur; UV-B, Ultra violet; Al, aluminum; Cu, cupper; Fe, Iron. The miRs down-regulated in stress are shown in Blue and miRs up-regulated in stress are shown in Red. The miRs for which the stress induced status is not available are represented by green.

The association of plant miRs with stress was first reported in 2004 (Sunkar and Zhu, 2004). Now there are a number of reports supporting the hypothesis for the function for miRs in the adaptive response to abiotic stress including drought (Liu et al., 2008b; Zhou et al., 2010), cold (Zhou et al., 2008), salinity (Liu et al., 2008a; Sunkar et al., 2008) and nutrient deficiency (Fujii et al., 2005). 1062 miRs have been reported to be differentially expressed in 35 different abiotic stress types in 41 plant species (Zhang et al., 2013b). The detailed list of these miRs is available as Supplementary Table 1. The comparative picture of stress-induced dis-regulations of Arabidopsis and rice miRs is compiled as Figure 3.

The survey of literature reveals that three major approaches have been employed for the identification and expression profiling of stress induced miRs. The first approach involves the classical experimental route that included direct cloning, genetic screening, or expression profiling. The second method involved computational predictions from genomic or EST loci and the third one employed a combination of both as it was based on the prediction of miRs from High Throughput Sequencing (HTS) data. Each of these was followed by experimental validations by northern analysis, PCRs or microarrays.

### Experimental approaches

Direct cloning was the principal and conventional method for the identification of miRs (Park et al., 2002; Reinhart et al., 2002). This method was of significant consideration as it was a sequence-independent approach where *a priori* knowledge of miR sequence was not required. Moreover, it provided more accuracy and efficiency by giving few false positives. Several related studies led to the establishment of different protocols for sRNA isolation and adaptor mediated synthesis of a cDNA library followed by their amplification and then cloning. The clones were screened and sequenced to identify the potential miRs (Llave et al., 2002; Reinhart et al., 2002; Sunkar and Zhu, 2004). Thus, it was portrayed as a time-consuming, low throughput, laborious, and expensive approach.

However, the first report indicating the role of miRs in plant responses to environmental stresses came from the sequencing and analysis of a library of sRNAs from Arabidopsis seedlings treated with cold, dehydration, salinity, and the plant stress hormone abscisic acid (ABA). It was observed that several miRs were up-regulated or down-regulated by the abiotic stresses (Sunkar and Zhu, 2004). This strategy was used to clone miRs from the mechanical stress-treated Populus plants (Lu et al., 2005). A majority of these miRs were predicted to target developmental- and stress/defense-related genes. In our lab, 39 new miR sequences were cloned from salt-stressed basmati rice variety. This study also provided evidence for a converging functional role of miRs in managing both abiotic and biotic stresses (Sanan-Mishra et al., 2009).

The importance of miRs in abiotic stress responses was also implicated by the fact that several mutants such as *hyl1, hen1*, and *dcl1* which are defective in miR metabolism, exhibited hypersensitivity to ABA, salt, and osmotic stresses (Lu and Fedoroff, 2000). Nonetheless, the direct evidence was provided by studies monitoring the down-regulation of miR398 expression in response to oxidative stresses, in Arabidopsis. It was later shown that miR398 targeted two Cu/Zn superoxide dismutase (CSD) transcripts, cytosolic CSD1, and chloroplastic CSD2, so stress induced reduction of miR398 was expected to improve plant tolerance. This theory was proved subsequently by analysis of transgenic lines under oxidative stress conditions (Sunkar et al., 2006).

Expression analysis by northern blot analysis revealed that miR395 and miR399 were involved in sulfate and inorganic phosphate starvation responses, respectively (Jones-Rhoades and Bartel, 2004; Fujii et al., 2005). Similarly, RNA gel blot analysis identified miRs induced by cold, ABA, dehydration, and high salinity in 2-week-old Arabidopsis seedlings (Sunkar and Zhu, 2004). The results indicated that Ath-miR393 was highly up-regulated whereas Ath-miR397b and Ath-miR402 were slightly up-regulated and Ath-miR389a.1 was down-regulated under all the stress treatments. Similarly low temperature stress condition induced the expression of Ath-miR319c but no increase in response to dehydration, NaCl or ABA (Sunkar and Zhu, 2004). These and related findings not only helped in interpreting the role of miRs during stress but unraveled the role of specific members of the miR family. A comprehensive study of Ath-miR398, revealed that the expression of miR398 precursors (with identical mature sequences) is increased under high temperature stress and that heat stress induces expression of Ath-miR398b to a much higher level than that of the Ath-miR398a,c (Guan et al., 2013). Similarly in rice, Osa-miR169g, was proven as the only drought stress induced member among the ABA responsive miR169 family (Zhao et al., 2007).

The variable expression patterns of the miRs in response to different stresses were captured by reverse transcription quantitative PCR (RT-PCR) in several plants including Arabidopsis (Jung and Kang, 2007; Reyes and Chua, 2007; Li et al., 2008; Liu et al., 2008a; Jia et al., 2009), rice (Liu et al., 2009), *Phaseolus vulgaris*, (Arenas-Huertero et al., 2009), sugarcane (Thiebaut et al., 2012), and poplar (Rossi et al., 2015). These methods captured the similarities and differences in expression profiles of conserved miRs across different plants (Zhou et al., 2010). This is exemplified by identified molecules like miR393 that is consistently up-regulated during drought stress in many plants such as Arabidopsis, Medicago, common bean, and rice (Sunkar and Zhu, 2004; Zhao et al., 2007; Arenas-Huertero et al., 2009). Whereas miR169 was found to be induced by drought and high salinity in rice (Zhao et al., 2009), but was down-regulated by drought stress treatment in Arabidopsis (Li et al., 2008). High-throughput expression profiling analysis through one-tube stem-loop RT-PCR quantified the relative expression levels of 41 rice miRs under drought, salt, cold, or ABA treatments (Ding et al., 2011).

The need for genome wide characterization of miR expression profiles established the microarray analysis as a useful tool (Garzon et al., 2006; Zhao et al., 2007). The microarray technology is a hybridization based and a relatively cost-effective assay that allows analysis of large numbers of molecules in parallel. The tiling path microarray analysis was used to identify 14 stress-inducible Arabidopsis miRs after screening 117 miRs under high-salinity, drought, and low-temperature stress conditions (Liu et al., 2008a; Zhang et al., 2008b). The results were further validated to provide evidence for cross-talk among the high-salinity, drought and low temperature stress associated signaling pathways (Liu et al., 2008a). Similar studies were performed to capture the expression patterns of miRs in response to Ultraviolet-B rays in Arabidopsis (Zhou et al., 2007), drought stress in rice (Zhao et al., 2007), cold stress in rice (Kang et al., 2010), cadmium stress in rice (Ding et al., 2011), and ABA and NaCl in *Populus tremula* (Jia et al., 2009).

The expression patterns also identified that tissue-specific regulation of miRs may be important for adaptation to stress. Under water deficit conditions, miR398a/b and miR408 were up-regulated in both roots and shoots of *Medicago truncatula* plant, but the increase was more pronounced in the shoots than in the roots. This was accompanied by the down-regulation of their corresponding targets, COX5b and plantacyanin, thereby suggesting that these miRs have a crucial role in regulation of plants responses against water deficiency (Trindade et al., 2010). In barley, miR166 was up-regulated in leaves, where as it was shown to be down-regulated in roots; and miR156a, miR171, and miR408 were induced in leaves, but unaltered in roots (Kantar et al., 2010).

The miR expression profiles were also used to compare the genotypic differences between varieties exhibiting contrasting stress sensitivities. Microarray profiles of salt-resistant and susceptible *Zea mays* identified 98 miRs belonging to 27 families (Ding et al., 2009). Zma-miR168 family members were induced in the salt-tolerant maize but suppressed in the salt-sensitive line. Interestingly this salt-responsive behavior of miR168 was found to be conserved in Maize and Arabidopsis (Liu et al., 2008a). miR microarray was also used to study drought-tolerant wild emmer wheat (*Triticum dicoccoides*) (Kantar et al., 2011), two cotton cultivars with high tolerance (SN-011) and high sensitivity (LM-6) to salinity (Yin et al., 2012) and for comparative analysis between drought-resistant and susceptible soybean (Kulcheski et al., 2011). A comparison of 12 salt-tolerant and 12 salt-susceptible genotypes in *Oryza sativa*, identified 12 polymorphic miR based simple sequence repeats (Mondal and Ganie, 2014). Only miR172b-SSR was different between the salinity stress tolerant and susceptible genotypes. The genotype-dependent miR profiles suggested that response of miRs to abiotic stresses varies among closely related genotypes with contrasting stress sensitivities. The result of this analysis showed that there was less diversity of miR genes in the tolerant as compared with susceptible cultivars (Mondal and Ganie, 2014).

### Computational predictions

The detection and validation of miRs by molecular cloning was supported by systematic approaches using computational techniques (Bonnet et al., 2004b). These approaches also complemented the experimental methods by identifying difficult to clone miR families such as miR395 and miR399 (Jones-Rhoades and Bartel, 2004; Adai et al., 2005) which were difficult to detect by experimental approaches due to their low expression levels. Computational predictions strategies have been quite useful in miR identification in various plant species such as Arabidopsis (Wang et al., 2004; Adai et al., 2005; Li et al., 2005b), rice (Li et al., 2005b; Zhang et al., 2005), maize (Zhang et al., 2006a, 2009b), tomato (Yin et al., 2008; Zhang et al., 2008b), foxtail millet (Khan et al., 2014), soybean (Zhang et al., 2008a), *Brassica napus* (Xie et al., 2007), apple (Gleave et al., 2008), grape (Carra et al., 2009), and some other plants (Zhang et al., 2005; Sunkar and Jagadeeswaran, 2008).

It had been verified that a majority of known miRs are evolutionarily conserved and are expected to have homologs or orthologs in other species. So search criteria allowed up-to three sequence mismatches while looking for conserved miRs in heterologous species. Using this approach 85 conserved sequences which were showing perfect match to miRs reported in miRBase (Release 19) were predicted from *Morus notabilis* tissues (Jia et al., 2014). Whereas in another study 35 miR families were identified in heat stressed *Brassica napus* by allowing two mismatches with *A. thaliana* miRs (Yu et al., 2012). Thus, the conserved sequence of plant miRs and other structural features were used for developing suitable strategies and rules for identifying and annotating (Discussed in Section The Influence of Bioinformatics Approaches on microRNA Nomenclature and Annotation) new miR genes (Lagos-Quintana et al., 2001; Reinhart et al., 2002; Floyd and Bowman, 2004; Wang et al., 2004; Adai et al., 2005; Zhang et al., 2006a; Lukasik et al., 2013). One of the early comprehensive computational analysis by Jones-Rhoades and Bartel (2004) systematically identified plant miRs and their regulatory targets that are conserved between Arabidopsis and rice. Using MIRcheck algorithm they predicted that the miRs could target mRNAs like superoxide dismutases (SOD), laccases, and ATP sulfurylases that are involved in plant stress responses. Such studies lead to identification of involvement of Ath-miR398 in the ROS pathway by targeting sites on Cu/Zn-SOD (Jones-Rhoades and Bartel, 2004; Sunkar and Zhu, 2004; Lu et al., 2005; Sunkar et al., 2005) A similar approach was used in miRFinder computational pipeline, to identify 91 conserved plant miRs in rice and Arabidopsis (Bonnet et al., 2004a).

Another strategy was based on the property of miRs to bind with perfect complementarity to their target transcripts (Laufs et al., 2004). In plant species where the target sequence was available the conserved miRs could be easily predicted by using 20 mer genomic segments with not more than two mismatches as *in silico* probes. This target-guided strategy was adopted to identify 16 families of drought stress-associated miRs from *Physcomitrella patens* (Wan et al., 2011).

The computational predictions also utilized the criteria for conservation of miR sequence and key secondary structure features of pre-miRs like their characteristic fold-back structure, thermodynamic stability etc. to predict new miRs (Berezikov et al., 2006). Seventy-nine putative miRs were identified in wheat using traditional computational strategy, out of which 9 were validated by northern blot experiments (Jin et al., 2008). Subsequently bioinformatics tools like miRAlign were developed based on the requirement of structural similarity and sequence conservation between new candidates and experimentally identified miRs (Wang et al., 2005). Though numerous miR profiles were generated by the computational algorithms, this was not found to be appropriate for species with less annotated genomes (Chen and Xiong, 2012).

The non-availability of complete genome annotation was overcome by employing the Expressed Sequence Tags (EST) database. These represented the true gene expression entities so they emerged as better indicators of dynamic expressions of the miR. A detailed study by identified 123 miRs from stress-induced ESTs of 60 plant species (Zhang et al., 2005). This study confirmed that irrespective of evolutionary divergence miRs are highly conserved in plant kingdom and miR genes may exist as orthologs or homologs in different species within the same kingdom (Weber, 2005; Zhang et al., 2006b). The EST database was also used to confirm some novel miRs identified earlier by computational strategies in citrus (Song et al., 2010) and peach (Zhang et al., 2012). In a recent study ESTs of abiotic stress treated libraries of *Triticum aestivum* were used to identify novel miRs in drought, cold, and salt stressed cDNA libraries by searching all mature sequences deposited in the miRBase (Release 19) (Pandey et al., 2013).

### High throughput sequencing

The recent development of HTS approaches has invoked a new era by allowing the sequencing of millions of sRNA molecules. The HTS techniques employ sequencing-by-synthesis (SBS) technology, which enable accessing the full complexity of sRNAs in plants. In addition, it provides quantitative information of the expression profiles, since the cloning frequency of each sRNA generally reflects its relative presence in the sample. The signature-based expression profiling method such as massively parallel signature sequencing (MPSS) has identified miRs that have thus far proven difficult to find by using traditional cloning or *in silico* predictions. Sequencing technologies are rapidly emerging as the favored alternatives to the microarray-based approaches, since direct measures of gene expression can be obtained through sequencing of random ESTs, SAGE, and MPSS. The expression patterns of the identified miR targets can then be followed in the transcriptome sequencing data to gain novel insights into plant growth and development and stress responses (Wang et al., 2010; Li et al., 2013). Though currently an expensive technique, it is expected that as the technology grows, it will become more affordable.

Complex computational algorithms are used to rapidly and rigorously sift through the HTS data for identification of putative miRs (**Figure 5**). These datasets have been very successful in identification of conserved miRs where the sequence is well maintained across plant species. The targets for these miRs can also be easily predicted using Parallel Analysis of RNA End (PARE) sequencing, where miR and its target mRNA have often nearly perfect complementarily (Rhoades et al., 2002; Bonnet et al., 2004b; Jones-Rhoades and Bartel, 2004). The HTS data also provided a useful source to hunt for the non-conserved or species-specific miRs based on the criteria of miR annotation (Discussed in Section The Influence of Bioinformatics Approaches on microRNA Nomenclature and Annotation).

This HTS approach was initially used to visualize the repertoire of sRNAs in Arabidopsis (Rajagopalan et al., 2006; Fahlgren et al., 2007), followed by investigation on the rice miR expression profiles in drought and salt stress responses (Sunkar et al., 2008). Later, Liu and Zhang identified 67 arsenite-responsive miRs belonging to 26 miR families from *Oryza sativa* (Liu and Zhang, 2012). Solexa sequencing was also used to identify conserved and novel miRs in *Glycine max* libraries from water deficit and rust infections (Kulcheski et al., 2011), cold responsive miRs in trifoliate orange, *Poncirus trifoliate*, (Zhang et al., 2014a), drought and salinity responsive miRs in *Gossypium hirsutum* (Xie et al., 2015), heat stress induced miRs in *Brassica napus* (Yu et al., 2012), and salt stressed miRs in *Raphanus sativus* (Sun et al., 2015). Regulation of miRs in response to various abiotic stresses was studied in Arabidopsis, under drought, heat, salt, and metal ions such as copper (Cu), cadmium (Cd), sulfur (S) excess or deficiency, using sRNA NGS libraries. The search for most profound changes in miR expression patterns identified that miR319a/b, miR319b.2, and miR400 were responsive to most of the stresses under study (Barciszewska-Pacak et al., 2015).

Comparative profiles of miR expression during cold stress among *Arabidopsis, Brachypodium*, and *Populus trichocarpa* revealed that miR397 and miR169 are up-regulated. This indicated the presence of conserved cold responsive pathways in all the species. Whereas the differences in the pathways was highlighted by miR172 which was up-regulated in Arabidopsis and *Brachypodium* but not in poplar (Zhang et al., 2009a). Opposing patterns of miR regulation in different plant species during cold stress were observed for miR168 and miR171. The miRs are up-regulated in poplar (Lu et al., 2008) and Arabidopsis (Liu et al., 2008a) but down-regulated in rice (Lv et al., 2010). Likewise the HTS analysis of salt stressed sRNAome identified 211 conserved miRs and 162 novel miRs, belonging to 93 families between *Populus trichocarpa* and *P. euphratica* (Li et al., 2013). Using the approach of comparative miR profiling followed by experimental validation, our group identified 59 Osa-miRs that show tissue-preferential expression patterns and significantly supplemented 51 potential interactive nodes in these tissues (Mittal et al., 2013).

HTS technology has also played a crucial role in identification and characterization of the miR targets with PARE or Degradome sequencing. This involves sequencing of the entire pool of cleaved targets followed by mapping of the miR-guided cleavage sites (Ding et al., 2012). In Populus, 112 transcripts targeted by 51 identified miRs families were validated by using degradome sequencing (Li et al., 2013). These are several reports which used HTS of sRNA pools and degradome analysis to identify targets of stress induced miRs such as, in maize (Liu et al., 2014), tomato (Cao et al., 2014), *Raphanus sativus* (Wang et al., 2014), Populus (Chen et al., 2015), rice (Qin et al., 2015), *Phaseolus vulgaris* (Formey, 2015), and barley (Hackenberg et al., 2015).

It has been shown that plant miRs also act by inhibiting mRNA translation (Brodersen et al., 2008; Lanet et al., 2009), therefore such targets tend to get overlooked during degradome sequencing. The HTS techniques are also being employed for sequencing the whole transcriptome pools to identify the miR targets in *Medicago* (Cheung et al., 2006), *Zea mays* (Emrich et al., 2007), and Arabidopsis (Weber et al., 2007). The combined strategy of sRNAs and mRNAs (transcriptome) sequencing enabled the identification of new genes, involved in nitrate regulation and management of carbon and nitrogen metabolism in Arabidopsis. This study identified miR5640 and its target, *AtPPC3*, leading to the preposition that the ${\text{NO}}_{3}^{-}$ responsive miR/target might be involved in modulating the carbon flux to assimilate nitrate into amino acids (Vidal et al., 2013).

## The influence of bioinformatics approaches on microRNA nomenclature and annotation

The *in silico* approaches have also played a dominant role in the identification of plant miRs and their targets. The advancement in molecular and computational approaches has not only resulted in the exponential growth in the discovery and study of sRNA biology but has also provided a deeper insight into the miR regulatory circuits. At the same time, they have been instrumental in defining and redefining the rules for annotating the miRs and their nomenclature.

**Figure 4:**
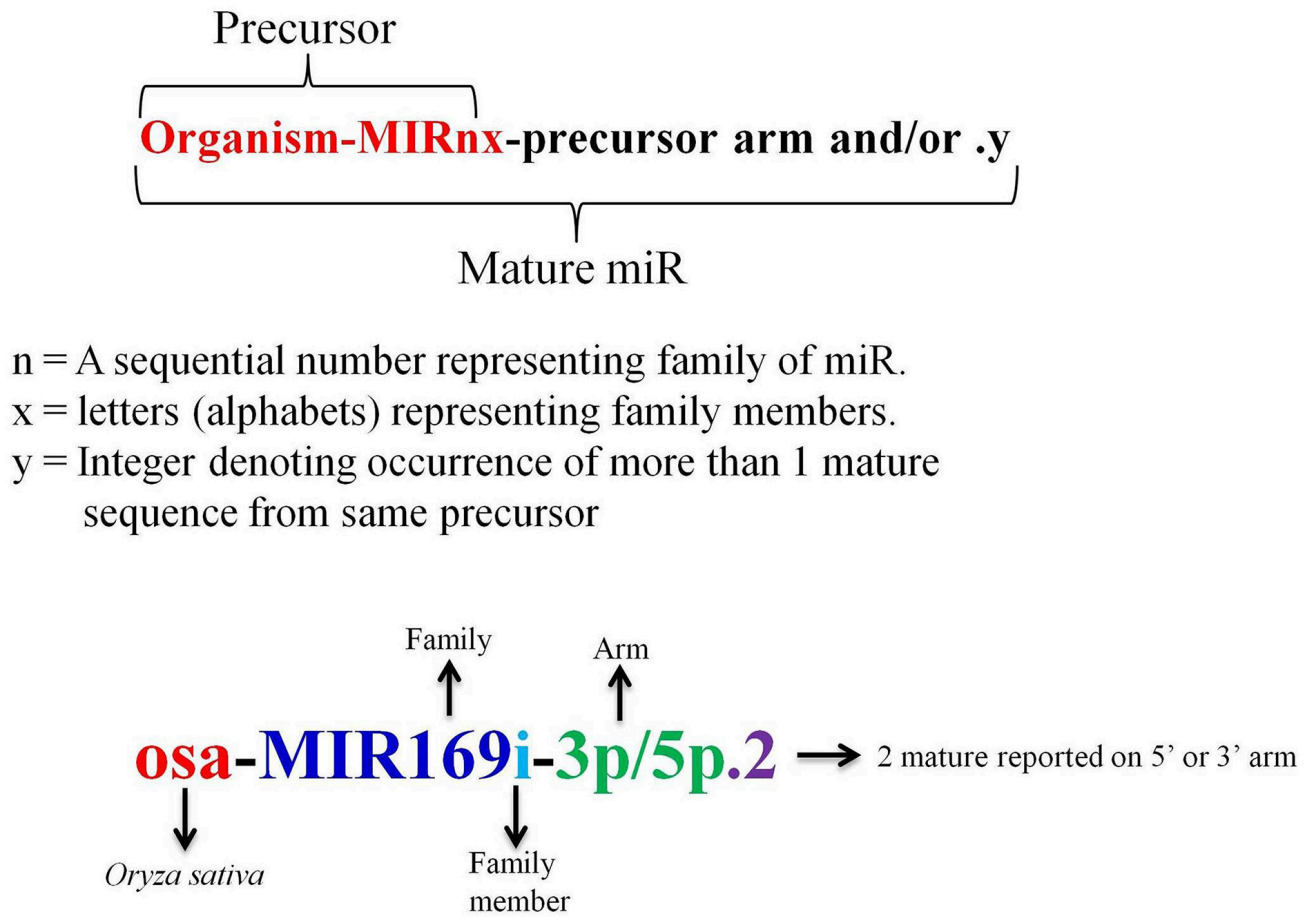
**Nomenclature schema of miRs**.

A miR registry system was adopted in 2004 to facilitate a complete and searchable place for the published miRs and to provide a systematic rule so that the new miRs can be assigned with a distinctive name prior to publication of their discovery (Ambros et al., 2003; Griffiths-Jones, 2004). In miRBase the nomenclature of miRs starts with initial 3 letters signifying the organism, followed by a number which is simply a sequential numerical identifier based on sequence similarity, suffixed by “miR,” trailed by alphabet letters which denotes the family member (Figure 4). It was later enforced that sequences showing homology within organisms and mature identical sequences coming from two or more different organism should be assigned the same family names (Meyers et al., 2008). Sequences with no similarity to previously reported sequence were considered novel and assigned next number in the series (Griffiths-Jones, 2004). It is observed that in miRBase *Medicago truncatula*, mtr-miR2592 is the largest miR family with 66 members, while in rice; the largest family is seen for Osa-miR395 with 25 members. The occurrence of more than 1 mature sequence from same precursor is designated by an integer followed by a dot at the end (Griffiths-Jones, 2004; Meyers et al., 2008). With the accumulation of HTS data and the experimental validation that both miR and miR^*^ of same precursor can be functional, it was decided to add a suffix of 3p and 5p at the end of the sequence to represent the presence of miR on 3′ or 5′ arm of stem loop precursor (Meyers et al., 2008).

The processing of biological information through bioinformatics tools and computational biology methods has now become crucial for elucidating complicated biological problems in genomics, proteomics, as well as in metabolomics. With the accumulation of huge sRNA sequencing datasets, it is almost impossible to analyze each and every sequence through direct experimental approaches. This has necessitated the role of bioinformatics tools and databases in analyzing and screening the huge data sets in a short time period, with minimum costs and without compromising on the specificity of analysis.

The primary criteria for annotation of plant miRs is the precise excision of a miR/miR^*^ duplex from the stem of a single-stranded, stem-loop precursor. Computational algorithms use these criteria to predict the RNA secondary structure for the sequences identified from the genomic DNA, transcript or ESTs. Subsequently the annotation rules are followed to distinguish a miR from the sRNA pool. The first set of guidelines for miR annotation was based on specific expression and biogenesis criteria (Ambros et al., 2003). The expression criteria included the identification by cloning and/or detection by hybridization and phylogenetic conservation of the miR sequence. While the biogenesis criteria included the presence of a characteristic hairpin structured precursor transcript, conservation of the precursor secondary structure and increased accumulation of a precursor in absence or reduction in Dicer activity (Ambros et al., 2003).

The advancement in sequencing technologies provided with highly sensitive techniques for obtaining the complete small RNA profiles that could distinguish between fragments differing by a single base. This also provided an excellent medium to search for known and novel miR family members, their precursors, and modified versions. The bioinformatics based analysis of HTS datasets, made it feasible to predict the entire set of miRs present in a RNA sample. This was also utilized to retrieve the information on expression profiles, putative target transcripts, the miR isoforms, and sequence variants of miRs through differential expression profiling under various conditions (Moxon et al., 2008; Addo-Quaye et al., 2009; Yang and Li, 2011b; Neilsen et al., 2012). Dedicated web servers like isomiRex (Sablok et al., 2013) are available online for identification of the sequence variants using HTS data.

With the development in computational tools and the availability of genomic sequences the rules were further refined to include characteristics that are both necessary and sufficient for miR annotation. It was proposed that the prediction criteria should include that the miR and miR^*^ are derived from opposite arm of same precursor such that they form a duplex with two nucleotide overhang at the 3′ end, base pairing of miR and miR^*^ should have less than four mismatched bases, the asymmetric bulges are minimum in size and frequency specifically in miR/miR^*^ duplex. sRNA-producing stem-loops that violate one of these criteria could still be annotated as miRs, provided that there is conclusive experimental evidence of precise miR/miR^*^ excision (Meyers et al., 2008). In continuation to the guidelines set by Ambros et al. (2003) it was recognized that conservation of miRs, assessed using either bioinformatics or direct experimentation, was still a powerful indicator of their functional relevance though it need not be necessary for annotation as many plant miRs lack homologs in other species. It was proposed that identification of a target is not necessary for miR annotation as targets could not be predicted for many of the less-conserved miRs or the predicted targets lacked experimental confirmation.

It is being observed that increased coverage of deep-sequencing results have resulted in capturing sequences of ever-lower abundance. This has made the identification of miRs even more challenging. A number of recent publications have attempted use additional criteria based on patterns of mapped reads (Hendrix et al., 2010). The consensus set of guidelines that have started to emerge lay importance to the presence of multiple reads with consistent processing of the 5′-end of the mature sequence preferably from several independent experiments. The mapped reads should not overlap other annotated transcripts as they may represent fragments of mRNAs or other known RNA types.

**Figure 5:**
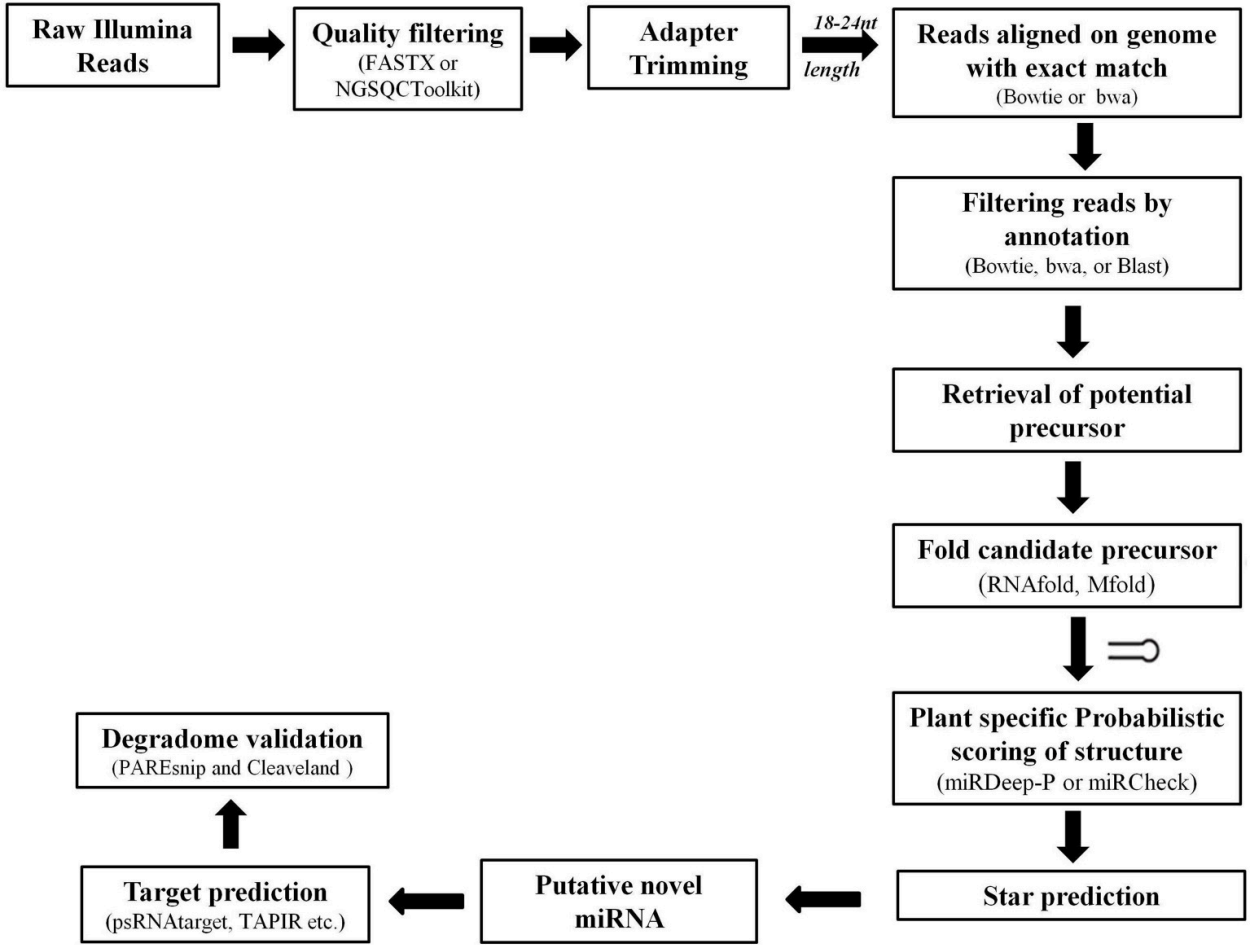
**Pipeline showing major steps for miR identification from high-throughput sequencing data**.

Various tools were developed based on the annotation guidelines to analyze the HTS data sets. The major steps adopted by various available tools for prediction of novel miRs and their target identification are discussed in Figure 5. Basically the sequenced reads are selected, based on the average quality score appended with each base, and subjected to 3′ adapter trimming. This can be achieved by designing specific scripts (using languages such as PERL) or by using various available tools such as NGSQC Toolkit (Patel and Jain, 2012), FASTX-Toolkit (Gordon and Hannon, 2010), CLC Genomics Workbench (Matvienko)^1^ etc. Next the reads with length of 18–24 nucleotides are selected and aligned to the corresponding genome of the plant species under consideration using tools such as bowtie, soap, and bwa. The aligned reads are then used to filter out sequences mapping with other sRNAs such as, tRNA, rRNA, sRNA, snRNA, snoRNA, and known miRs. The remaining reads are used to retrieve the potential precursors from the reference genome and their secondary structure is predicted. Excellent softwares like Mfold (Zuker, 2003), RNAfold (Denman, 1993) etc. are freely available and have been useful in identifying the appropriately folded structures. Then these candidate precursors are evaluated on the basis of the annotation criteria (Meyers et al., 2008). The expression profiles of identified known and novel miRs from sequence pools are achieved by calculating the number of times a unique read occurred in the entire sRNA pool and normalized against total reads. Reads Per Million (RPM) for each sequence occurring in each sample is most common way to achieve the normalized expression of each sequence. RPM = (Actual read count/total number of reads in sample) × 1,000,000) (Motameny et al., 2010).

## microRNA repositories

The study of miR and their targets by analyzing the sRNA and transcriptome sequences is greatly facilitated by the availability of numerous freely accessible tools and databases, which can be used by experimental researchers without any specialization in bioinformatics. The various web-based tools and databases available for the prediction and analysis of plant miRs and their targets are listed in Tables 1, 2, respectively. Each of these is based on different algorithms and methodologies and has their respective strengths and shortcomings. However, the major limitation in most of these techniques is the requirement for a known sequence and the search for a conserved hairpin loop structure (Unver et al., 2009). To overcome these limitations, Kadri et al. (2009) developed the Hierarchical Hidden Markov Model (HHMM) that employs region-based structural information of pre-miRs without relying on phylogenetic conservation. It obtains the secondary structures on the basis of minimum free energy and then classifies the sequence with HHMM (Kadri et al., 2009). Some of the popularly used tools are discussed below.

**Table 1:** **Major Plant databases providing information on the miR and their targets**.

**Name**	**Description**	**Link**	**References**
miRBase	Searchable database of published miR sequences and annotation	http://www.mirbase.org	Griffiths-Jones, 2004; Kozomara and Griffiths-Jones, 2010; Griffiths-Jones et al., 2008; Kozomara and Griffiths-Jones, 2014
deepBase	Database for annotating and discovering small and long ncRNAs (miRs, siRNAs, piRNAs) from high-throughput deep sequencing data.	http://deepbase.sysu.edu.cn/	Yang and Qu, 2012
PMRD	Database involving miRs and their target genes, especially model plants and major crops	http://bioinformatics.cau.edu.cn/PMRD/	Zhang et al., 2010
PNRD	It is an updated version of PMRD	http://structuralbiology.cau.edu.cn/PNRD/index.php	Yi et al., 2015
PMTED	Plant miR Expression Database	http://pmted.agrinome.org/	Sun et al., 2013
Plant MPSS	Measure's the expression level of most genes (including sRNA and their targets) under defined conditions and provide information about potentially novel transcripts. with the help of public HTS data	http://mpss.udel.edu/	Nakano et al., 2006
miRTarBase	The experimentally validated miR-target interactions database	http://mirtarbase.mbc.nctu.edu.tw/	Hsu et al., 2010
*Rfam*	A resource for predicted miR targets and expression	http://rfam.xfam.org/	Burge et al., 2013
ARMOUR	A Rice miRNA: mRNA Interaction Resource	http://armour.icgeb.trieste.it/	Unpublished

**Table 2:** **Major tools for analyzing plant miRs and their targets**.

**Name**	**Description**	**Link**	**References**
PASmiR	A literature-curated database for miR molecular regulation in plant response to abiotic stress	http://pcsb.ahau.edu.cn:8080/PASmiR/	Zhang et al., 2013b
isomiRex	Web portal to identify miRs and their isoforms as well as differential expression of NGS datasets	http://bioinfo1.uni-plovdiv.bg/isomiRex/	Sablok et al., 2013
CLC genomics Workbench	Analyze, compares and visualizes NGS data	http://www.clcbio.com/products/clc-genomics-workbench/	
miRTarBase	The experimentally validated miR-target interactions database	http://mirtarbase.mbc.nctu.edu.tw/index.php	Hsu et al., 2010
MIRFINDER	Computational pre-miR prediction tool	http://www.bioinformatics.org/mirfinder/	Bonnet et al., 2004b
Targetfinder	Predicts small RNA targets in a sequence database using a plant-based scoring metric	http://carringtonlab.org/resources/targetfinder	
mirCheck	A PERL script designed to identify RNA sequences with secondary structures similar to plant miRs	http://bartellab.wi.mit.edu/software.html	Jones-Rhoades and Bartel, 2004
findmiRNA	Predicts potential miRs within candidate precursor sequences that have corresponding target sites within transcripts	http://sundarlab.ucdavis.edu/mirna/	Adai et al., 2005
MicroInspector	A web tool for detection of miR binding sites in a RNA sequence	http://bioinfo.uni-plovdiv.bg/microinspector/	Rusinov et al., 2005
RNAhybrid	Calculates a minimal free energy hybridization of RNA sequence(s) and miR(s)	http://bibiserv2.cebitec.uni-bielefeld.de/rnahybrid/	Krüger and Rehmsmeier, 2006
CleaveLand	A pipeline for using degradome data to find cleaved small RNA targets	http://axtell-lab-psu.weebly.com/cleaveland.html	Addo-Quaye et al., 2009
TAPIR	Target prediction for Plant miRs	http://bioinformatics.psb.ugent.be/webtools/tapir/	Bonnet et al., 2010
psRNATarget	A plant sRNA target analysis server	http://plantgrn.noble.org/psRNATarget/	Dai and Zhao, 2011
miRanalyzer	miR detection and analysis tool for next-generation sequencing experiments	http://bioinfo5.ugr.es/miRanalyzer/miRanalyzer.php	Hackenberg et al., 2011
PmiRKB	Plant miR knowledge base includes the miRs of two model plants, Arabidopsis and rice. Four major functional modules, SNPs, Pri-miRs, MiR-Tar and Self-reg, are provided	http://bis.zju.edu.cn/pmirkb/	Meng et al., 2011b
miRDeep-P	A computational tool for analyzing the miR transcriptome in plants	http://faculty.virginia.edu/lilab/miRDP/	Yang and Li, 2011b
C-mii	A tool for plant miR and target identification	http://www.biotec.or.th/isl/c-mii	Numnark et al., 2012b
Semirna	Searching for plant miRNAs using target sequences	http://www.bioinfocabd.upo.es/semirna/	Muñoz-Mérida et al., 2012
UEA sRNA Workbench	A suite of tools for analysing and visualizing NGS datasets	http://srna-workbench.cmp.uea.ac.uk/	Stocks et al., 2012
mirTool	A comprehensive web server providing detailed annotation information for known miRs and predicting novel miRs that have not been characterized before	http://centre.bioinformatics.zj.cn/mirtools/	Wu et al., 2013
miRPlant	An Integrated Tool for Identification of Plant MiR from RNA Sequencing Data	http://www.australianprostatecentre.org/research/software/mirplant	An et al., 2014
MTide	An integrated tool for the identification of miR-target interaction in plants	http://bis.zju.edu.cn/MTide/	Zhang et al., 2014b

### miRcheck

This is an algorithm written in the form of a PERL script for identifying 20 mers having potential to encode plant miRs. The tool requires input of a putative hairpin sequences and their secondary structures. The presence of candidate 20 mer sequences is then searched within the hairpin to predict potential plant miR. This algorithm was first used for identifying conserved miRs in Arabidopsis and rice (Jones-Rhoades and Bartel, 2004).

### UEA sRNA Workbench

It is a comprehensive tool for the complete analysis of sRNA sequencing data and provides the convenience of using the facilities provided by different tools in one place. Its Graphical User Interface (GUI) makes it easy to use for researchers, do not needs any prior knowledge of computer programming (Moxon et al., 2008). It can be downloaded and installed locally, and it also has a web-based facility of doing the same analysis in form of UEA sRNA toolkit which is freely accessible. Table 3 lists all the available tools at UEA sRNA Workbench.

**Table 3:** **Tools available on UEA sRNA Workbench and their functions in analyzing the sRNA sequencing data**.

**Tool**	**Function**	**References**
Adapter removal	Removes the adapter sequence	Moxon et al., 2008
Filter	It filters already annotated sRNA (rRNA, tRNA. snRNA, snoRNA, miRNA etc) data	Moxon et al., 2008
Sequence alignment	Allows alignment of short reads to the genome	Moxon et al., 2008
CoLIde	It defines a locus as a combination of regions sharing same expression profiles, present in close proximity on genome	Mohorianu et al., 2013
miRCat	Predicts miRs from HTS data without requiring the precursor sequence	Moxon et al., 2008
miRProf	Determines normalized expression levels of sRNAs matching to known miR in miRBase	Moxon et al., 2008
PAREsnip	Finds target of sRNA using degradome data.	Folkes et al., 2012
SiLoCo	Compares expression patterns of sRNA loci among different samples	Moxon et al., 2008
ta-si Prediction	Trans-acting RNA prediction, by identifying 21nt characterstic of ta-siRNA loci by using sRNA dataset and respective genome	Moxon et al., 2008
RNA/Folding annotation	Predicts the secondary structure of RNA sequences and annotates it by highlighting up to 14 comma seperated short sequences	Moxon et al., 2008
VisSR	Used for sequence visualization	Moxon et al., 2008

### TAPIR

This is an online web server for prediction of targets of plant miRs. It can characterize miR-targets duplexes with large loops which are usually not detectible by traditional target prediction tools. The prediction results are driven by a combination of two different algorithms. The first one is the fast and canonical FASTA local alignment program which cannot detect duplexes with large number of bulges and/or mismatches (Pearson, 2004) and second one is RNAhybrid (Krüger and Rehmsmeier, 2006) for detection of miR-mRNA duplexes (Bonnet et al., 2010). Though it is a good option for miR target prediction but is not preferred as the users face problem in analyzing large datasets on the online server.

### CLC Genomics Workbench

It is a commercial software developed by QIAGEN that offers Quality Check (QC) and pre-processing of NGS data. Although it is a good tool for preprocessing of NGS data but it focuses more on other genomic areas such as *de novo* assembly and it doesn't provides the facility to process the sRNA data for miR and target identification. In relation to the sRNAs it has been majorly used in initial steps of quality filtering, adapter trimming and calculating abundances of sRNA libraries. It can also generate genome alignments by using standalone blast search. The workbench provides an interactive visualization to the differential expression and statistical analysis of RNA-Seq and sRNA data.

### C-mii

It uses a homology-based approach for plant miR and target identification. The tool aligns known miRs from different plant species to the EST sequences of the query plant species using blast homology search. The aligned sequences are allowed to fold in to the characteristic hairpin loop structures to identify the putative miRs. The predicted miR sequences are further used for identifying perfect or nearly perfect complimentary sites on the input transcript sequences to identify the putative targets. The tool has a unique feature of predicting the secondary structures of the miR-target duplexes. The identified targets can be annotated further by searching their functions and Gene Ontologies (GO) (Numnark et al., 2012a). It provides user friendly GUI, and is easily downloadable hence it can be easily used for analyzing large datasets. However, the major limitation lies in the search and availability of homologous sequences, so it cannot be used to analyze the NGS datasets.

### miRDeep-P

It is a collection of PERL scripts that are used for prediction of novel miRs from deep sequencing data. It was developed by incorporating the plant-miR specific criteria to miRDeep (Friedländer et al., 2008). Its pipeline utilizes bowtie for sequence alignments and RNAfold for secondary structure prediction of putative precursors. The remaining steps such as extracting potential precursor sequences and identification of putative novel miR is regulated by specific scripts (Yang and Li, 2011a). Although it is a specialized tool for identification of plant miRs, but does not has a GUI interface. So the user needs to work through command line for its execution, which warrants knowledge on PERL scripting.

### CleaveLand

It is a general pipeline, available as a combination of PERL scripts, for detecting miR-cleaved target transcripts from degradome datasets (Addo-Quaye et al., 2009). It can be executed by a single command and requires input of degradome sequences, sRNAs, and an mRNA database to yield an output of cleaved targets. The pipeline runs in command mode and requires the co-installation of several dependencies such as PERL, R, samtools, bowtie, RNAplex etc.

### ARMOUR

The accumulation of sequencing data has generated the need for a comprehensive and integrated database of miR:mRNA, expression profile information and target information. Our group has developed ARMOUR database (A Rice miRNA: mRNA Interaction Resource) that consolidates extensive datasets of rice miRs from various deep sequencing datasets for examining the expression changes with respect to their targets. Development of such interactomes for different plant species shall provide a valuable tool to biologists for selecting miRs for further functional studies.

## Perspectives

miRs are an extensive class of endogenous, small regulators of gene expression in the numerous developmental and signaling pathways. There is ample evidence for the role of miRs in abiotic stress mediated genomic changes that result in attenuation of plant growth and development. The different experimental approaches have identified the intriguing expression profiles of miRs in distinctive tissues and/or stages of development. The regulation of miR expression also varies between the domesticated plant species and their wild relatives. Sequence-based profiling along with computational analysis has played a pivotal role in the identification of stress-responsive miRs, although these results require independent experimental validations. sRNA blot and RT-PCR analysis have played an equally important part in systematically confirming the profiling data. The identification of putative targets for these miRs has provided robust confirmation of their stress responsiveness. This has also enabled quantification of their effect on the genetic networks, such that many of the stress regulated miRs have emerged as potential candidates for improving plant performance under stress. However, so many efforts are still required for in-depth analysis of the miR modulation of each gene product induced by abiotic stress(es) and its interacting partners. This requires development of reliable and rigorous assays for firm characterization of the spatio-temporal regulation of these miRs under stress conditions. The potential of computational biology needs to be tapped for performing an extensive comparison of miR expression profiles among agriculturally important crops during environmental stress conditions to tap key target nodes that need to be modulated for improving crop tolerance to environmental stress. The development and integration of plant synthetic biology tools and approaches will add new functionalities and perspectives in the miR biology to make them relevant for genetic engineering programs for enhancing abiotic stress tolerance.

### Conflict of interest statement

The authors declare that the research was conducted in the absence of any commercial or financial relationships that could be construed as a potential conflict of interest.

## Footnotes

^1^Matvienko, M. CLC Genomics Workbench.

## Supplementary material

Supplementary Table 1 **List of all abiotic stress associated plant miRs**.
